# The Death Penalty

**Published:** 1890-10

**Authors:** 


HALL'S
JOURNAL OF HEALTH

TRUTH DEMANDS NO SACRIFICE; ERROR CAN MAKE NONE.

Vol. 37. OCTOBER, 1890. No. 10.

### THE DEATH PENALTY.

For more than a year past there has been in the great State of New York, the most populous, if not the most advanced of the family of States composing our nationality, a contentious struggle over the life of one poor misguided human being, who, under the law of his Commonwealth, had incurred the death penalty. He had been tried and convicted of willful murder, under circumstances so atrocious, as to leave in his behalf little room for sympathy, and little, indeed, would there have been, had the execution of the death sentence proceeded in the customary manner. But prior to his trial and conviction, the law, from motives of humanity, be it conceded, prescribed a new and hitherto untried means of taking human life, under its sanction. Strangulation by the halter was superseded by the deadly shock of the electric current, which had proved so instantaneously fatal to man and beast, in divers cases of accidental contact. A long course of litigation followed the sentence, wherein every legal objection that could be raised to the law, was put forth and judicially passed upon by authorized tribunals, adversely to the condemned, who was eventually made ready for the ordeal through which he was to pass, and which, to say the least, was more or less an experiment.

The spectacle of a powerful State in arms against a single member, with appliances, devices and complex machinery, wherewith to take his life, is any thing but a satisfactory one to contemplate. Even if the doomed had been a dumb brute without sense or feeling beyond the instinctive dread of impending evil, the situation would have drawn to it unwonted sympathy. The terrible details of preparation, and the more terrible execution, to which the hapless victim lent a needed hand, are fresh in the minds of our readers. Had he resisted, what a revolting scene would have been the consequence! As it was, sturdy men sickened and fainted, and were borne away.

What is to be the outcome of these wretched proceedings? Were they not enough to cause all men to pause and enquire into the right and justice of enforcing in the present, the cruel and inhuman practices of an ancient and semi-barbarous people, whose sole protection against the persistent evil doer was his destruction? The same necessity does not inhere in respect to the civilized communities of our day. There are prisons and strongholds now, where none were before, wherein the malefactor may be kept secure against a repetition of his crime. What more is required? What further purpose of the State can be justifiably maintained? Human laws are very imperfect at the best. They deal only with the fact of crime, and scarcely ever reach behind it and investigate the provocation. Many a convicted criminal is morally superior to his accuser, as well as to those that pass upon his crime, ay, and the judge that pronounces his sentence, be it what it may. Then, too, we have repeated instances of the trial, conviction and suffering, even unto death, of persons subsequently shown to have been wholly innocent. Let us be done, once and for all, with this lawful taking of human life. It is inhuman; it is unjust; it is something we cannot restore to the innocent, though the proof of innocence be never so conclusive. Let the demand of the people for reform be voiced in no uncertain terms. The time for it is ripe, and the occasion is at hand. If need be, let impregnable strongholds be built wherein to confine exclusively the malicious destroyers of life.

But, says one, they might be freed by an official pardon. And why not, should they deserve it? Would you deprive one of life for want of confidence in the integrity of your Governor? If so, deprive the Executive of this prerogative in respect to life sentences, and trust to tribunals of justice to rectify errors by holding inquests in the nature of new trials, when the justice of such a measure is made to appear. It is the theory of the law that for every wrong there is a remedy. What greater folly than to destroy the remedy and thus compel the wrong to go unrighted.

> "The greatest attribute of Heaven is MERCY;
> And 'tis the crown of justice, and the glory,
> Where it may kill with right, to save with pity."

